# The Tale of Nine Belgian Health Ministers and a Multi-level Fragmented Governance System: Six Guiding Principles to Improve Integrated Care, Responsiveness, Resilience and Equity; A Response to the Recent Commentaries

**DOI:** 10.34172/ijhpm.2022.7848

**Published:** 2023-01-31

**Authors:** Sara Van Belle, Joris Michielsen, Tom Cornu, Monika Martens, Bruno Marchal

**Affiliations:** ^1^Department of Public Health, Institute of Tropical Medicine, Antwerp, Belgium.; ^2^Department of Family Medicine and Population Health (FAMPOP), Faculty of Medicine and Health Sciences, University of Antwerp, Antwerp, Belgium.

 In 2021, Martens et al published a paper presenting a comprehensive stakeholder analysis of three policy junctures in Belgium’s quest for improved integrated care (IC). The authors noted that Belgium’s “*2014 partial decentralisation of healthcare has created fragmentation of decisive power which undermines efforts towards IC.*”^1^ In five commentaries, academics, stakeholder representatives and frontline practitioners provided a kaleidoscopic view on the barriers hampering policy initiatives on IC.^2-6^ We first comment on the commentaries before taking a step back from the subject of IC and proposing guiding principles towards defragmenting the Belgian health system.

 An obvious first step to make Belgium’s multi-level governance system work is to redefine the responsibilities as clear and unambiguous as possible, in which process principles of hierarchy and subsidiarity matter.^6^ However, any such change will remain *high politics* in Belgium and thus heavily contested: one could as much argue in favour of a re-centralisation as for a full decentralisation. This makes meso-level mechanisms,^2^ including collaborative governance and distributed leadership,^7^ attractive. Such approaches that are rooted in complex adaptive systems make sense given the social and thus complex nature of any health system. Yet, other complexity principles apply as well. This is illustrated by the coronavirus disease 2019 (COVID-19) pandemic, which put a harsh spotlight on the structural weaknesses of the Belgian health system, but which also catalysed important changes. In Belgium, the Primary Healthcare Zones were fertile grounds for local innovation in the fight against the Corona virus.^6^ Any approach that stimulates systematic learning, such as pilot projects^5^ or learning networks,^3^ would have been useful if only they would have been fully institutionalized and applied beyond specific diseases. Indeed, complexity theory points out that in situations of high uncertainty, decentralization of decision-making, information sharing and effective support to actors confronted by the problem stimulate innovation. Central actors should define the objectives, implement fail-safe pilots, scale up successful innovations and stop harmful actions. This requires a system capable of systematic learning.^8^ In governance terms, adaptive governance^9^ is called for. Rooted in social-ecological systems theory, local adaptive governance is the foundation for responsive and equitable health systems. Furthermore, adaptive governance is central to resilient systems^10^ - now more than ever a priority for any health system.

 While the comments point to approaches required at local, regional and national level, we argue that central to any effort to improve Belgium’s health system is a redrawing of its governance structure. We present six interlinked principles.

1. **Sense-making in building a shared long-term vision**. To start defining a long-term vision, actors should move away from the currently entrenched positions.^6^ All actors would need to accept that governance reform is essential for any health policy initiative to succeed. Powerful uniting narratives include the persistent health inequity and the need to make the health system both sustainable and resilient in order to address the effects of the climate crisis and other shocks.
2. **Foster the mind shifts required for health governance reform. **Thesocial values and societal dispositions underlying health systems and system reforms constitute a key element of health system complexity.^11^ Values and principles, such as social accountability in which patients participate, responsiveness, equity, sustainability, equitable resilience and bottom up intersectoral action, indicate the need for more inclusive negotiated spaces than the current medico-legal concertation model provides.^4^ In Belgium, the Primary Care Zone could be a gamechanger in that it provides room of maneuver for local actors to create and implement a common vision in a context-sensitive way.
3. **In a polycentric field, meta-governors are required**. In Belgium’s multi-level, polycentric federal system, there is a need for clear allocations of mandates, roles and responsibilities.^6^ Yet, more important is the designation of meta-governors, who are responsible to coordinate and hold all actors accountable.^12^ The meta-governors are neutral, stable and operating beyond short-term and party-political goals and agendas. This requires rethinking the role and composition of agencies such as the National Institute for Health and Disability Insurance or the creation of new platforms.
4. **Clarify and strengthen accountability**. The lack of a “*strong integrative force*”^5^ and the opaqueness of the Belgian health governance system need to be actively managed in order to increase accountability towards the citizens, a key outcome of a well-functioning governance system. This is a responsibility of the meta-governors and it should be backed up by a strong legislative frame. Specific attention needs to be paid to give an active voice to groups who are currently underserved.^13^
5. **Pro-actively manage power dynamics at the local level.** In a predominant medico-centred culture,^4^ the composition and capabilities of the overarching Care Council (*ZorgRaad )* of the Primary Care Zone is crucial. Adequate funding should enable a well-resourced team in terms of skill mix, information systems and management capacity so that the Care Councils not only play their coordination and support role, but also take up a local meta-governor role and initiate innovations.
6. (**Co-)create a health management information and learning system** with and for all stakeholders, including patients and citizens in general, and healthcare and social care workers. Linking the local, regional and national level, such system should buttress decision-making processes at all levels.^5^ Belgium has been playing catch up compared to other countries since the end of the 1990s and needs to speed up its programme in order to ensure accountability and policy learning.

These principles would easily fit in a realist evaluation approach^14^ that seeks to elicit and test the programme theory underpinning such reforms. Its full development could bring together policymakers, stakeholder representatives and academics in a joint sense-making exercise.^2^ This exercise could not only clarify the stakeholder positions and expectations, but more importantly would identify essential conditions for the successful reconstruction of Belgium’s ineffective health governance system.

 It is now up to the political authorities to institutionalize and incorporate agility in the policymaking processes, institutional structures and management strategies so that these would valorize bottom-up and adaptive ways of working, while providing a strong central oversight. This should happen rather sooner than later.

## Ethical issues

 Not applicable.

## Competing interests

 Authors declare that they have no competing interests.

## Authors’ contributions

 SVB wrote the manuscript, BM edited the manuscript, MM, TC, and JM commented on the manuscript.
